# Supplementary material for: Inoculation with Mycorrhizal Fungi and Irrigation Management Shape the Bacterial and Fungal Communities and Networks in Vineyard Soils
Source: Microorganisms. 2021 Jun 11;9(6):1273. doi: 10.3390/microorganisms9061273 (PMC8230719; doi:10.3390/microorganisms9061273)
Supplement: Supplementary file 1 [file microorganisms-09-01273-s001.zip › Supplementary material.pdf]

Supplementary material:

Figure S1: Location of the treatment plots in the Merlot vineyard.

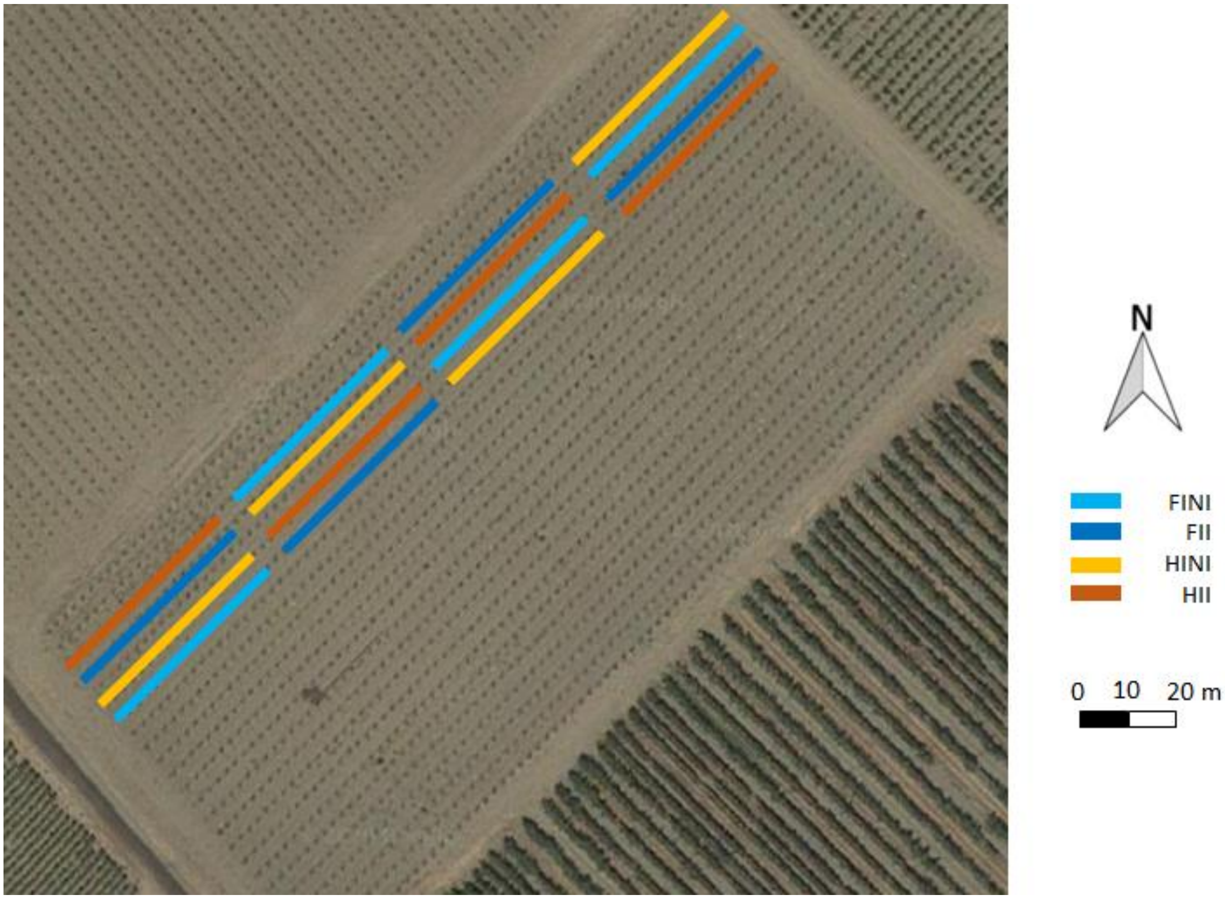

Figure S2: Number of OTUs shared among the different treatments (NT, FINI, FII, HINI and HII plots) for bacteria (A) and fungi (B) kingdoms. The numbers in the shaded overlapping zones indicate how many of the OTUs of the total number of OTUs (non shaded-non overlapping number) were shared among sample types.

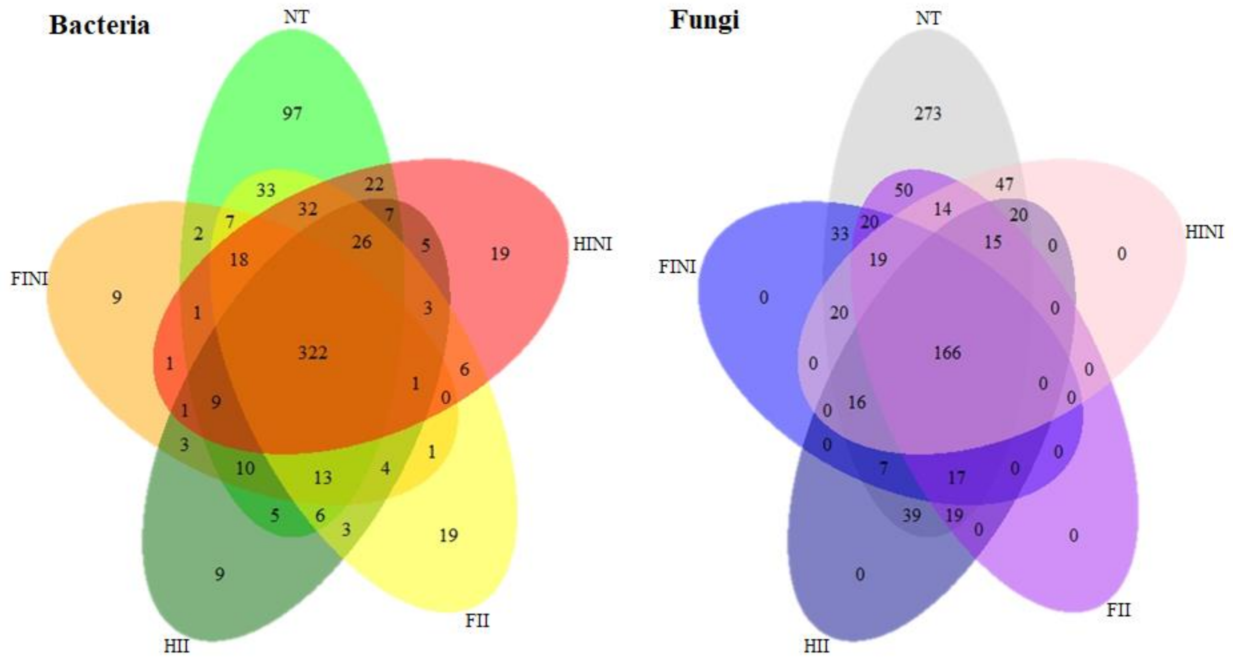

Figure S3: Relationships between the Shannon (A) and inverse of Simpson (B) indexes for bacterial and fungal communities found in plots before (NT) and after treatments, different irrigation amounts (FI, Full Irrigated or HI, Half Irrigated), and AMF inoculation (I, inoculated; or NI, non-inoculated). For each treatment, straight lines correspond to the linear regression lines and the 95% confidence interval.

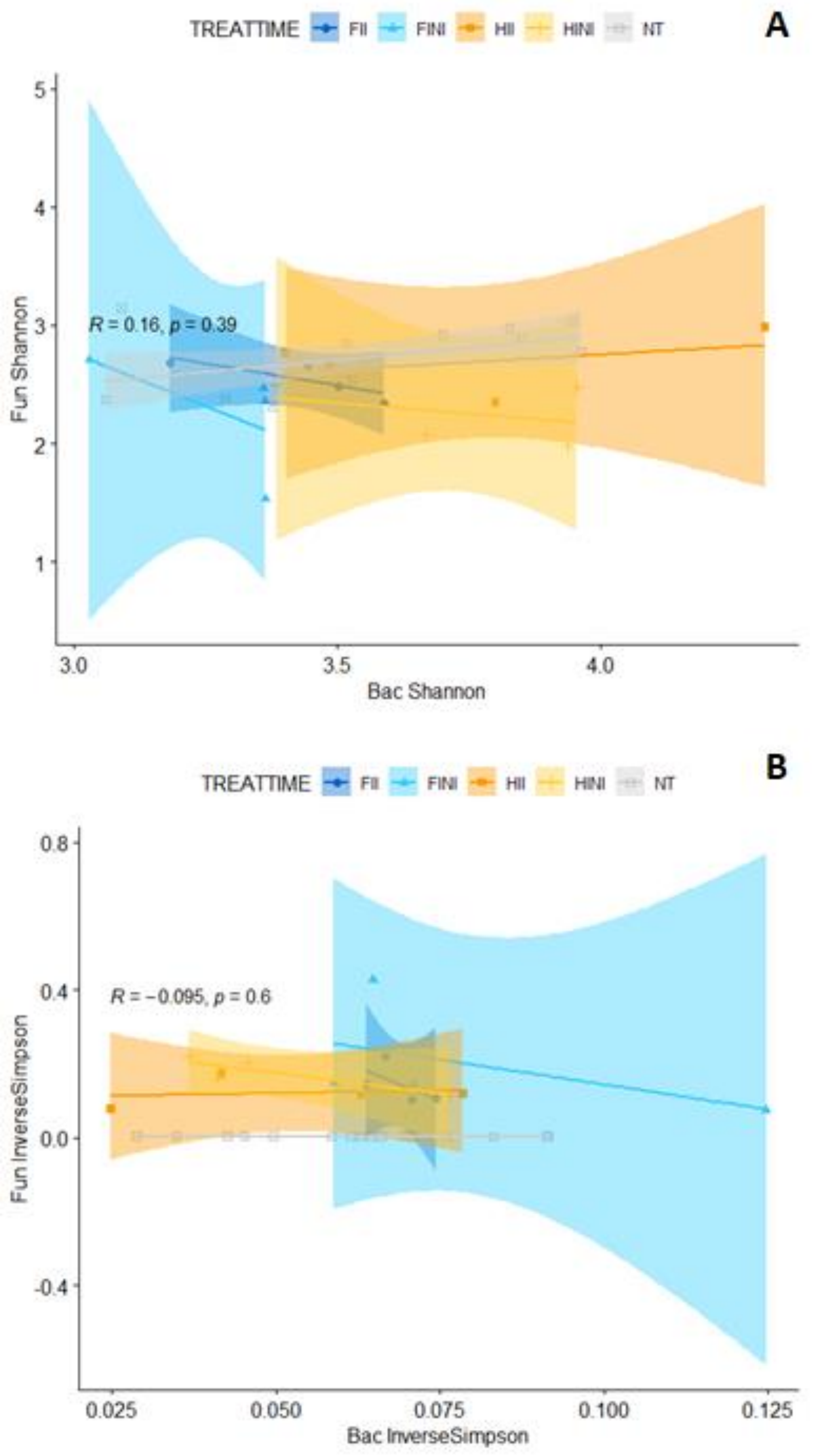

Figure S4. Heat tree with the taxonomy hierarchy of Proteobacteria phylum. Size and colors of nodes and edges are correlated with the abundance of organisms in each treatment. Green and brown were used to present higher or lower abundance, when comparing two conditions based on the different irrigation amounts (FI, Full Irrigated or HI, Half Irrigated), and AMF inoculation (I, inoculated; or NI, non-inoculated).

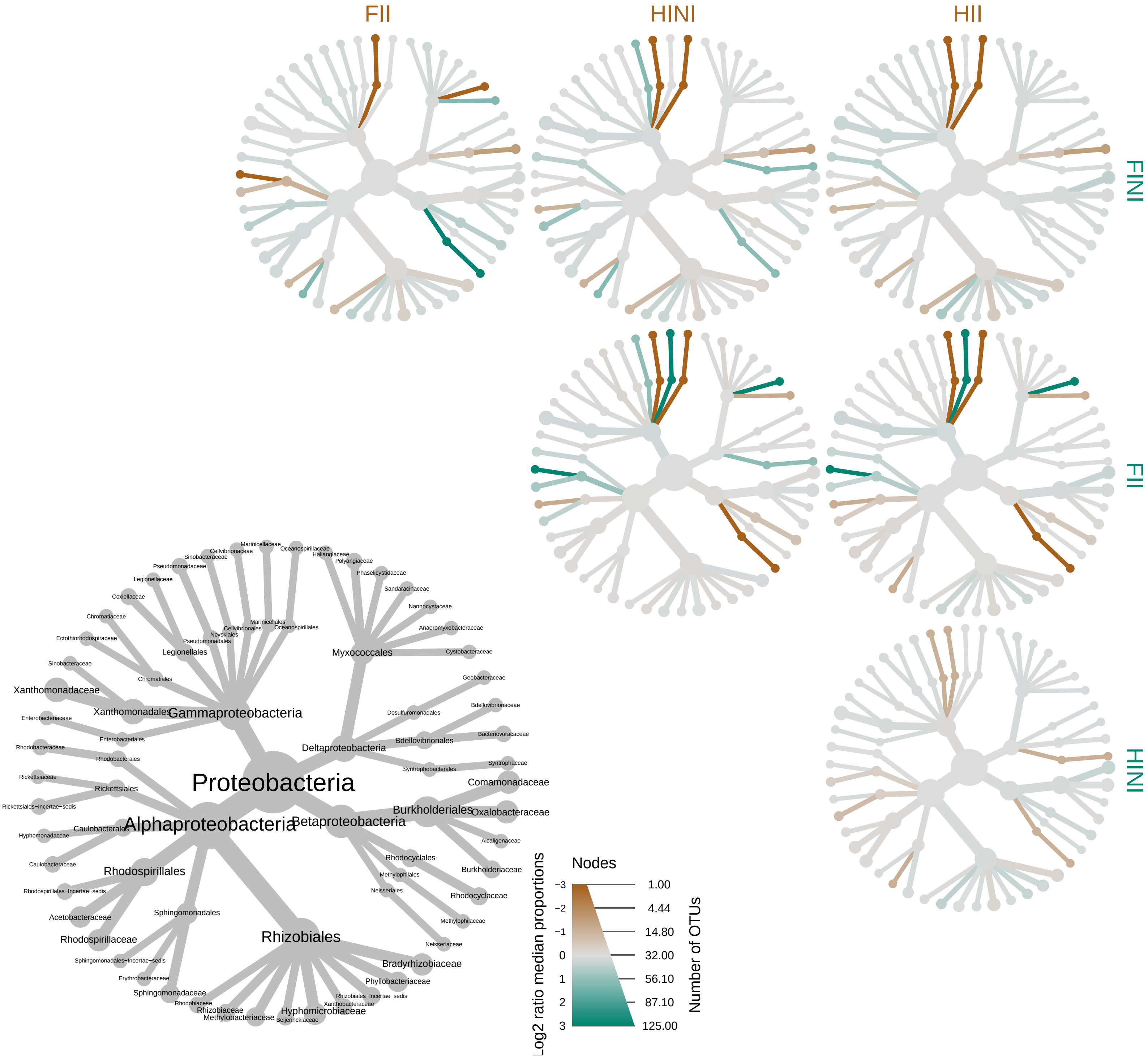

Figure S5. Heat tree with the taxonomy hierarchy of Actinobacteria phylum. Size and colors of nodes and edges are correlated with the abundance of organisms in each treatment. Green and brown were used to present higher or lower abundance, when comparing two conditions based on the different irrigation amounts (FI, Full Irrigated or HI, Half Irrigated), and AMF inoculation (I, inoculated; or NI, non-inoculated).

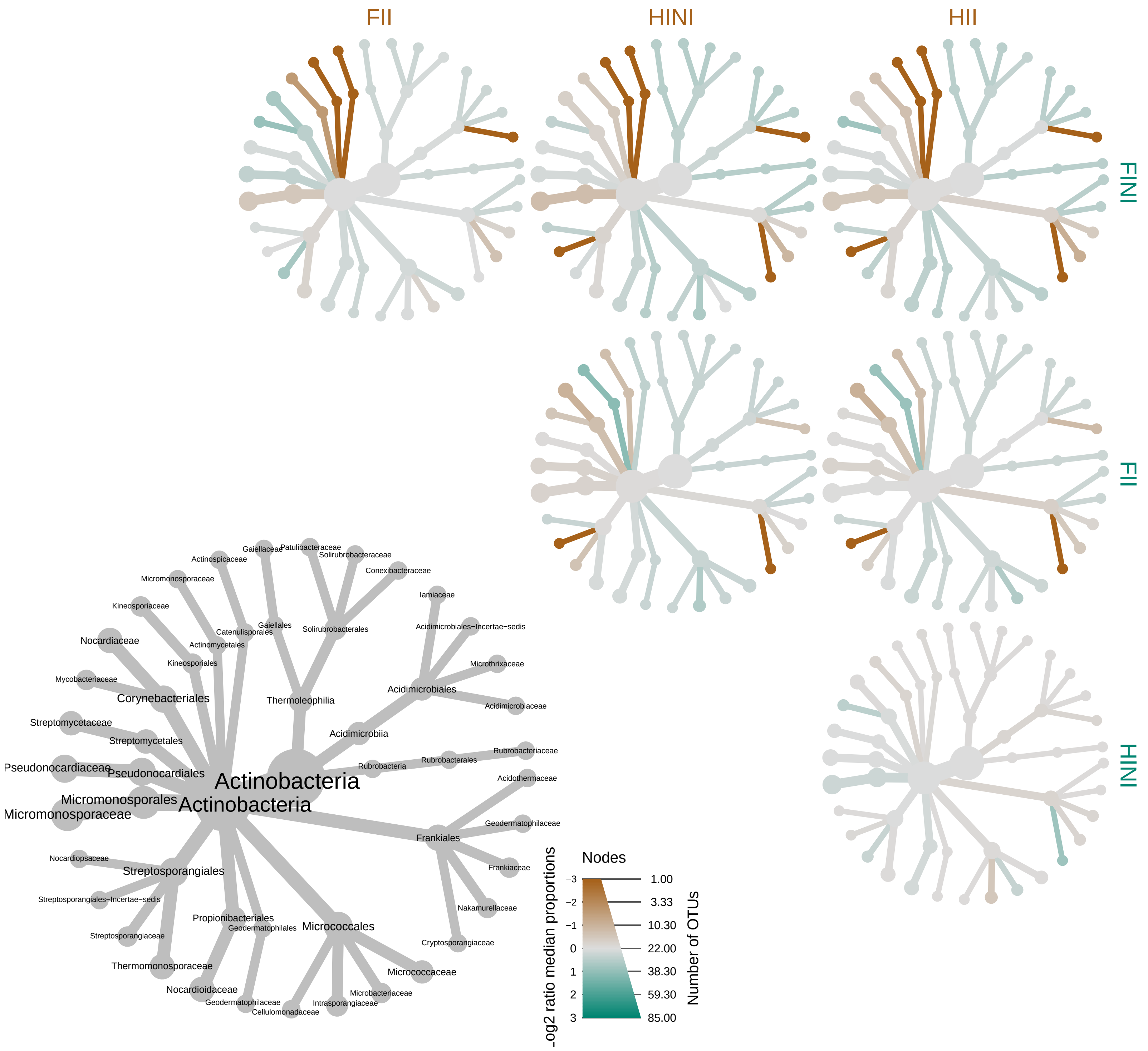

Figure S6. Heat tree with the taxonomy hierarchy of Firmicutes phylum. Size and colors of nodes and edges are correlated with the abundance of organisms in each treatment. Green and brown were used to present higher or lower abundance, when comparing two conditions based on the different irrigation amounts (FI, Full Irrigated or HI, Half Irrigated), and AMF inoculation (I, inoculated; or NI, non-inoculated).

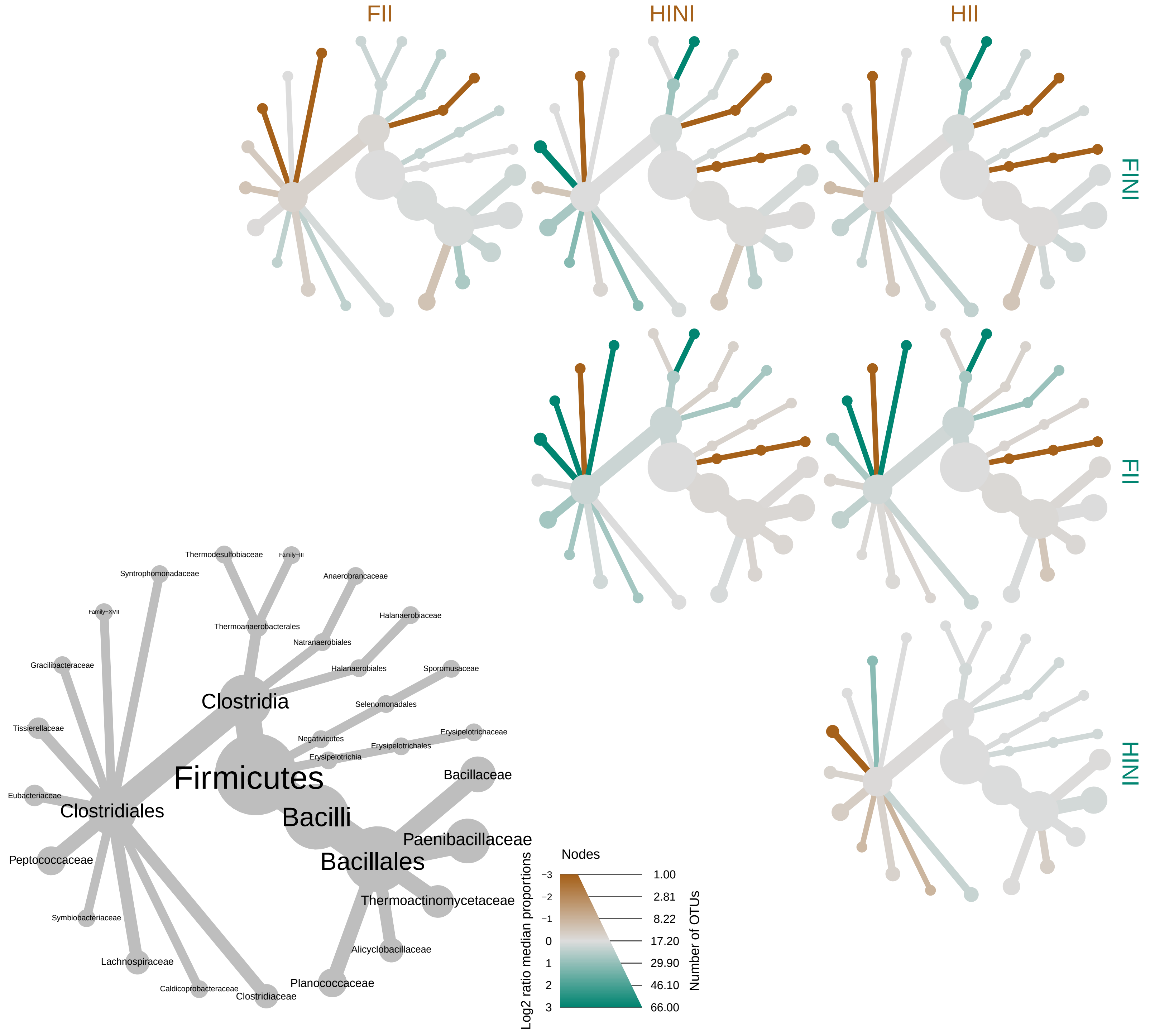

Figure S7. Heat tree with the taxonomy hierarchy of less abundant bacteria phyla. Size and colors of nodes and edges are correlated with the abundance of organisms in each treatment. Green and brown were used to present higher or lower abundance, when comparing two conditions based on the different irrigation amounts (FI, Full Irrigated or HI, Half Irrigated), and AMF inoculation (I, inoculated; or NI, non-inoculated).

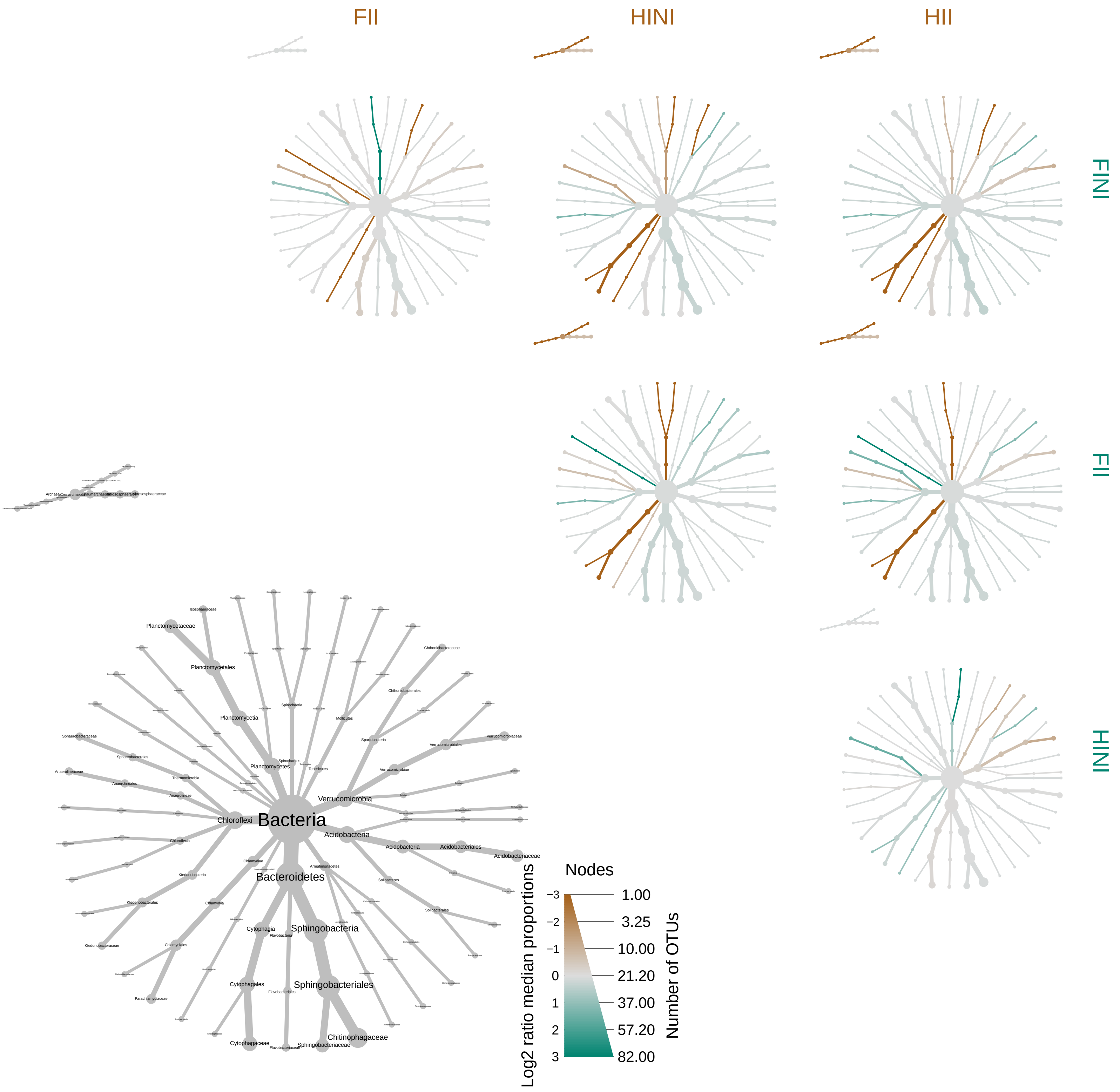

Figure S8. Heat tree with the taxonomy hierarchy of Ascomycota phylum. Size and colors of nodes and edges are correlated with the abundance of organisms in each treatment. Green and brown were used to present higher or lower abundance, when comparing two conditions based on the different irrigation amounts (FI, Full Irrigated or HI, Half Irrigated), and AMF inoculation (I, inoculated; or NI, non-inoculated).

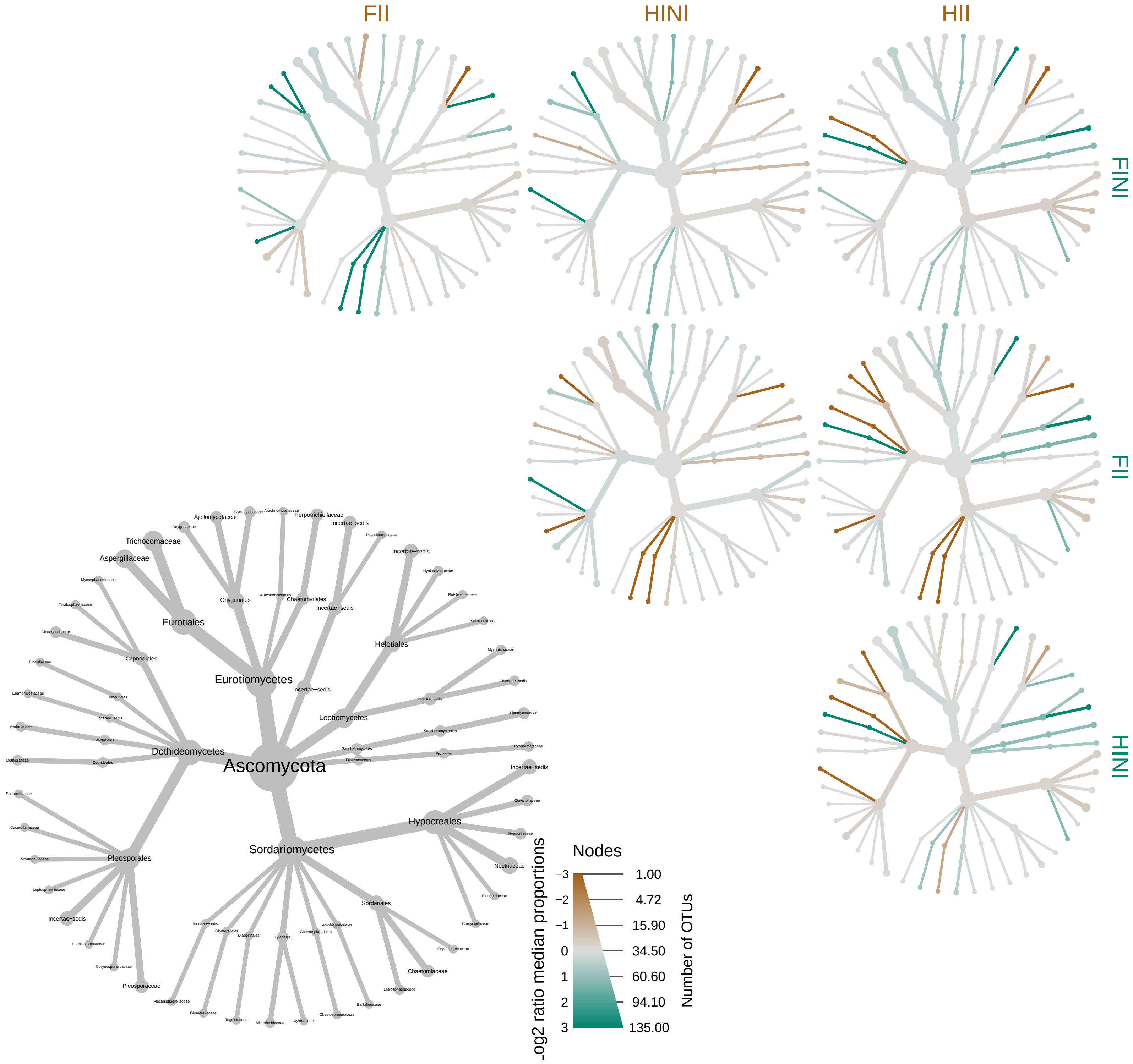

Figure S9. Heat tree with the taxonomy hierarchy of Basidiomycota phylum. Size and colors of nodes and edges are correlated with the abundance of organisms in each treatment. Green and brown were used to present higher or lower abundance, when comparing two conditions based on the different irrigation amounts (FI, Full Irrigated or HI, Half Irrigated), and AMF inoculation (I, inoculated; or NI, non-inoculated).

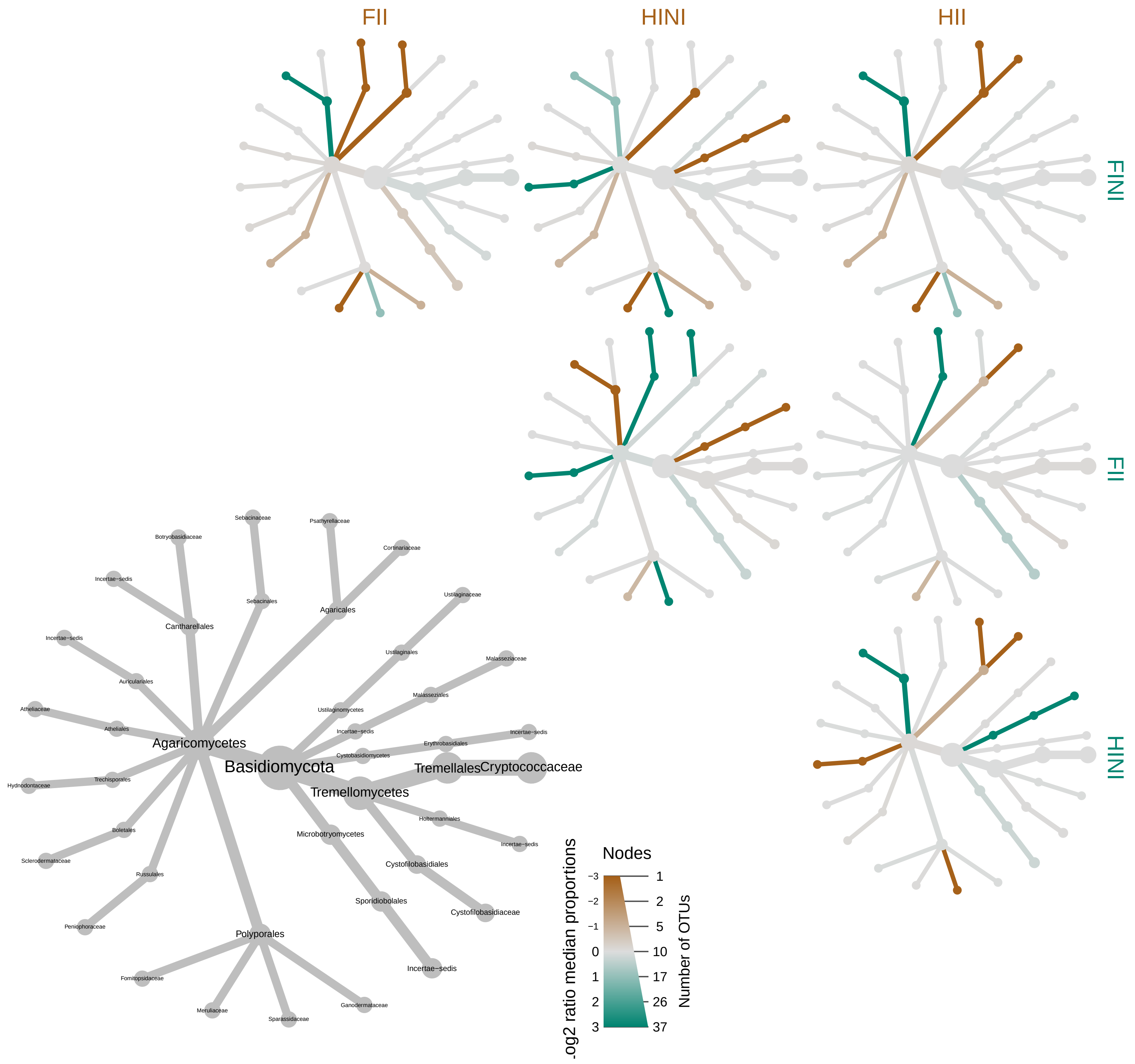

Figure S10. Heat tree with the taxonomy hierarchy of Zygomycota phylum. Size and colors of nodes and edges are correlated with the abundance of organisms in each treatment. Green and brown were used to present higher or lower abundance, when comparing two conditions based on the different irrigation amounts (FI, Full Irrigated or HI, Half Irrigated), and AMF inoculation (I, inoculated; or NI, non-inoculated).

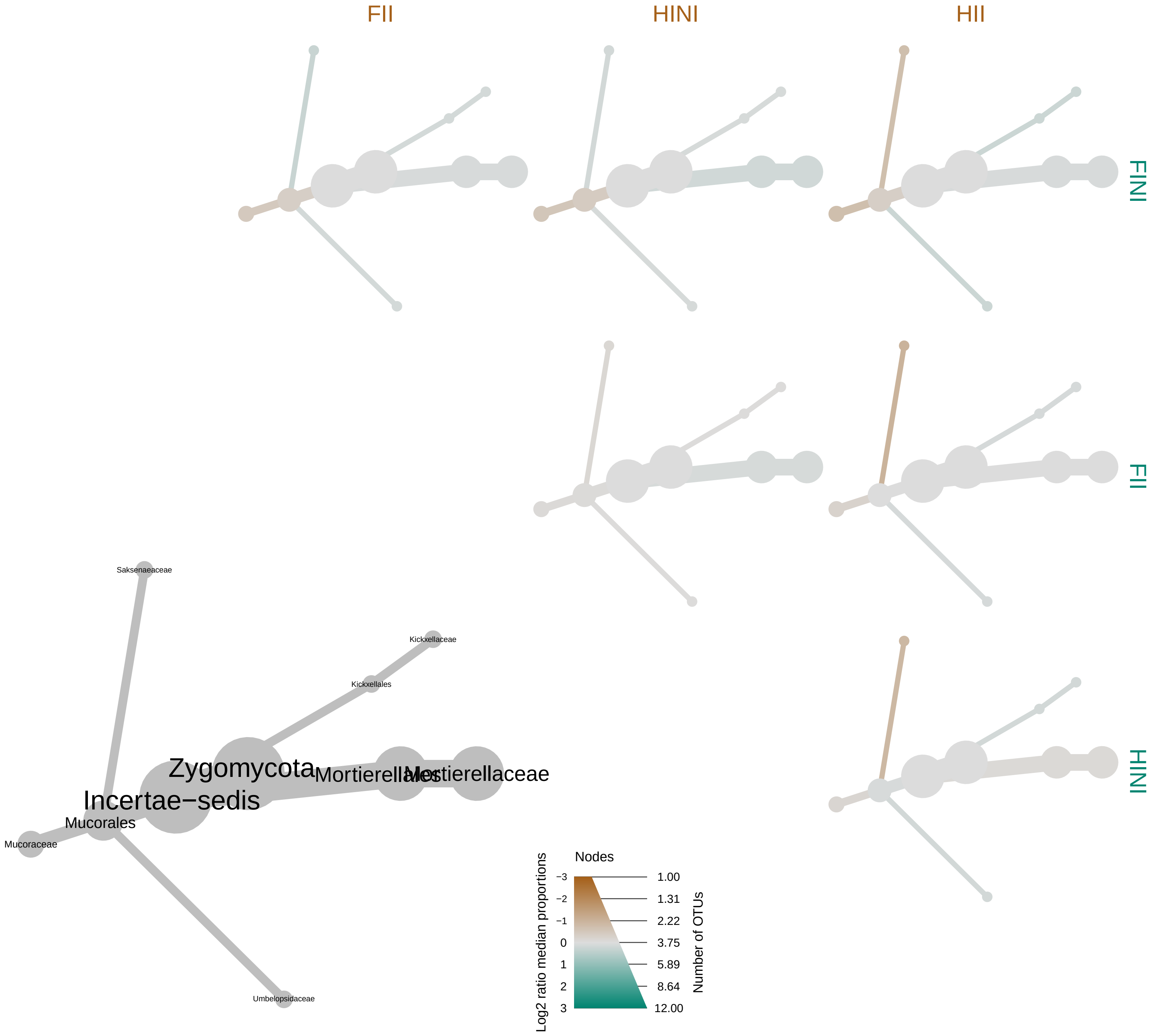

Table S1: Relationships between the Shannon and inverse of Simpson indices for bacterial and fungal communities within each not treated (NT) or treated plots subjected to different irrigation amounts (FI, Full Irrigated or HI, Half Irrigated), and AMF inoculation (I, inoculated; or NI, non-inoculated). R values, t values, degrees of freedom (df) and p-values of the regression study (Pearson correlation) conducted are reported.

Treatment	R	t	df	p-value
Shannon entropy				
**NT**	0.469	1.984	14	0.067
**FINI**	-0.580	-1.006	2	0.420
**FII**	-0.815	-1.992	2	0.185
**HINI**	-0.367	-0.558	2	0.633
**HII**	0.400	0.617	2	0.600
Inverse of Simpson				
**NT**	-0.443	-1.850	14	0.085
**FINI**	-0.524	-0.871	2	0.476
**FII**	-0.605	-1.076	2	0.395
**HINI**	-0.846	-2.245	2	0.154
**HII**	0.166	0.238	2	0.834

Table S2: Separation of phylum (A) or class (B) bacterial abundances between treatments by Kenward–Roger method and Tukey's p-value adjustment (P ≤ 0.05). Different letters within column, indicate significant differences as affected by not treated (NT) or subjected to different irrigation amounts (FI, Full Irrigated or HI, Half Irrigated), and AMF inoculation (I, inoculated; or NI, non-inoculated) and their combinations.

Table S2A:

	Contrast groups with Tukey's p value adjustment					LMEM	
**Phylum**	**NT**	**FINI**	**FII**	**HINI**	**HII**	F	p value
Acidobacteria	a	ab	b	ab	ab	3.03	0.032
Actinobacteria	b	ab	b	a	ab	4.8	0.003
Armatimonadetes	a	a	a	a	a	1.96	0.128
Bacteroidetes	ab	b	ab	ab	a	4.14	0.009
Chloroflexi	ab	ab	b	a	ab	3.42	0.019
Firmicutes	b	a	a	a	a	19.26	≤ 0.0001
Gemmatimonadetes	a	b	b	ab	b	10.41	≤ 0.0001
Planctomycetes	a	a	a	a	a	1.72	0.169
Proteobacteria	b	a	a	c	b	14.17	≤ 0.0001
Verrumicrobia	a	a	a	a	a	1.53	0.219
Others	b	b	b	a	ab	7.99	≤ 0.0001

Table S2B:

	Contrast groups with Tukey's p value adjustment					LMEM	
**Class**	**NT**	**FINI**	**FII**	**HINI**	**HII**	F	p value
Actinobacteria	b	ab	b	a	ab	4.42	0.006
Alphaproteobacteria	b	a	a	c	b	9.53	≤ 0.0001
Bacilli	b	a	a	a	a	17.45	≤ 0.0001
Betaproteobacteria	ab	a	a	c	bc	7.96	≤ 0.001
Gammaproteobacteria	b	b	b	a	ab	9.54	≤ 0.0001
Gemmatimonadetes	a	b	b	ab	b	10.41	≤ 0.0001
Planctomycetia	a	a	a	a	a	1.78	0.157
Rubrobacteria	a	a	a	a	a	1.8	0.153
Solibacteres	a	ab	b	ab	ab	3.03	0.032
Spartobacteria	a	a	a	a	a	1.64	0.193
Thaumarchaeota	a	b	b	a	a	40.18	≤ 0.0001
Thermoleophilia	a	a	a	a	a	1.19	0.331
Others	ab	b	b	a	ab	5.03	0.003

Table S3: Separation of phylum (A) or class (B) fungal abundances between treatments by Kenward–Roger method and Tukey's p-value adjustment ($P \leq 0.05$). Different letters within column, indicate significant differences as affected by not treated (NT) or subjected to different irrigation amounts (FI, Full Irrigated or HI, Half Irrigated), and AMF inoculation (I, inoculated; or NI, non-inoculated) and their combinations.

Table S3A:

	Contrast groups with Tukey's p value adjustment					LMEM	
**Phylum**	**NT**	**FINI**	**FII**	**HINI**	**HII**	F	p value
Ascomycota	a	ab	ab	b	ab	4.36	0.007
Basidiomycota	b	a	a	a	a	72.51	≥0.0001
Zygomycota	c	ab	a	bc	b	16.59	≥0.0001
Others	a	b	b	b	b	319.63	≥0.0001

Table S3B:

	Contrast groups with Tukey's p value adjustment					LMEM	
**Class**	**NT**	**FINI**	**FII**	**HINI**	**HII**	F	p value
Agaricomycetes	a	b	ab	ab	ab	4.26	0.008
Dothideomycetes	a	ab	ab	b	ab	5.07	0.003
Eurotimycetes	a	a	a	a	a	1.46	0.241
Leotiomycetes	a	c	ab	b	ab	7.98	≤ 0.001
Pezizomycetes	a	a	a	a	a	2.18	0.093
Saccharomycetes	a	b	ab	ab	b	6.11	0.001
Sordariomycetes	a	ab	b	b	b	15.06	≤ 0.0001
Tremellomycetes	b	a	a	a	a	42.67	≤ 0.0001
Others	a	b	b	c	bc	36.67	≤ 0.0001

Table S4: Canonical correspondence analysis (CCA) for bacterial communities. ANOVA table of the CCA model (A), Eigenvalues and their contribution to the scaled Chi-square (B). Scores for species in the first two components (highest scores are shown), species scaled proportioned to eigenvalues (C).

Table S4A:

ANOVA

	Df	ChiSquare	F	Pr(>F)
Model	6	0.22765	0.906	0.608

Table S4B:

Importance of components

	CCA1	CCA2	CCA3	CCA4	CCA5	CCA6
Eigenvalue	0.1558	0.0287	0.01408	0.01318	0.01167	0.004231
Proportion explained	0.2576	0.04747	0.02328	0.0218	0.01931	0.006998
Cumulative proportion	0.2576	0.30512	0.3284	0.3502	0.36951	0.376504

Table S4C:

Species scores

	CCA1	CCA2	CCA3	CCA4	CCA5	CCA6
Nitrososphaera sp.	0.90311	0.051796	0.05354	0.12737	0.005261	-0.04755
Jatrophihabitans sp.	0.840039	-0.17079	-0.14341	-0.23653	-0.20954	-0.08596
Actinophytocola sp.	0.816335	-0.05015	-0.02503	0.072079	-0.0816	0.115352
Pseudonocardia sp.	0.803977	0.070511	-0.06399	-0.088	0.043903	-0.01812
Geodermatophilus sp.	0.795144	-0.06155	-0.04005	-0.0704	-0.31164	-0.00122
Actinomycetospora sp.	0.783389	-0.05301	-0.03739	-0.06676	-0.07548	-0.00228
Rugosimonospora acidiphila	0.763093	0.249127	-0.05172	-0.10548	-0.00558	-0.03235
Micromonospora hermanusense	0.757079	-0.07651	-0.12834	-0.24113	0.123897	-0.07726
Pilimelia sp.	0.742563	0.162	-0.05302	-0.11957	-0.05318	-0.02341
Lysobacter sp.	0.741922	-0.9376	0.27309	0.35681	0.06597	0.127134
Afipia sp.	0.736507	-0.23968	-0.02602	-0.01671	-0.29163	0.172745
Actinomadura bangladeshensis	0.730014	0.105852	-0.16461	-0.3963	-0.04963	-0.12888
Cellulomonas xylanilytica	0.727358	-0.36363	-0.12005	-0.21129	-0.55435	0.068473
Mycobacterium sp.	0.69934	-0.10373	-0.06341	-0.47121	-0.11255	-0.12158
Sporocytophaga sp.	0.694234	-0.25704	-0.06803	-0.23426	-0.10568	0.173353
Roseiflexus sp.	0.692224	-0.02525	-0.06411	0.05317	0.027705	0.089307
Blastococcus sp.	0.682404	-0.16904	-0.07267	-0.12735	-0.2557	-0.05435
Actinoallomurus liliacearum	0.681606	0.055553	-0.08196	-0.19411	-0.24573	-0.01392
Chitinophaga sp.	0.651481	-0.19698	-0.04486	-0.31888	-0.20564	0.149745
Opitutus sp.	0.634364	-0.21628	-0.04987	-0.34872	-0.10618	-0.0653
Pseudonocardia halophobica	0.625778	-0.1957	-0.23561	-0.35158	-0.20011	0.087913
Adhaeribacter sp.	0.624527	-0.70378	0.102099	0.186241	-0.01492	0.113118
Streptosporangium sp.	0.619618	0.028435	-0.0078	0.063166	-0.1801	0.028091

Streptomyces atratus	0.610858	0.270358	-0.08153	0.053402	0.113615	0.088858
Crossiella sp.	0.605013	-0.1129	0.234968	0.412531	-0.143	0.03732
Acidothermus sp.	0.604694	0.145956	0.032827	0.097312	-0.12328	0.019451
Pseudomonas brassicacearum	0.599419	-0.44257	0.101634	0.171433	-0.21706	-0.04127
Chryseolinea sp.	0.584017	-0.23373	0.082954	0.08548	-0.49614	-0.3828
Flavobacterium sp.	0.576424	-0.3924	-0.07892	-0.24644	-0.12032	0.0838
Sediminibacterium sp.	0.575512	-0.24451	0.006759	-0.13939	-0.30003	-0.16434
Actinoallomurus sp.	0.568872	0.103537	0.038472	-0.09071	-0.02741	-0.0593
Iamia sp.	0.561461	0.069445	-0.05372	-0.32416	-0.1957	0.015219
Marmoricola sp.	0.546627	-0.16358	-0.29893	0.003933	-0.45004	0.088858
Streptomyces sp.	0.541773	0.122691	0.017125	0.063244	-0.187	0.00441
Streptomyces phaeochromogenes	0.539556	0.078553	-0.24487	-0.77926	-0.22819	0.237075
Acidiphilium sp.	0.516372	-0.23848	0.017026	0.157383	-0.2564	-0.01408
Rhodopirellula sp.	0.514133	-0.32655	0.028747	0.02222	-0.12991	0.078482
Reyranella sp.	0.489423	0.043458	-0.1318	-0.31596	-0.14176	0.029237
Cupriavidus basilensis	0.450702	-0.58379	0.251971	0.191967	0.276559	0.117796
Labrys sp.	0.438376	-0.00789	-0.14218	-0.14215	0.048079	-0.25277
Polycladomyces sp.	0.437634	-0.10789	0.124592	-0.22934	-0.30697	0.089599
Nitrospira sp.	0.432062	0.051167	-0.02968	-0.01631	0.172367	-0.03902
Acidicaldus sp.	0.426277	-0.03105	-0.12241	-0.0801	-0.25062	-0.09681
Kaistobacter sp.	0.422394	-0.64032	0.102711	-0.01616	0.046888	0.08476
Geobacter sp.	0.41423	-0.52195	0.035951	-0.13136	-0.1508	0.114173
Sandaracinus sp.	0.411317	-0.4303	-0.0215	-0.18697	-0.13011	-0.07674
Arthrobacter cereus	0.401652	0.168511	0.044562	0.039195	0.165427	0.0745
Caldilinea sp.	0.400044	-0.11916	-0.01428	0.076314	-0.19153	-0.00879
Pirellula sp.	0.39959	-0.03753	-0.04231	-0.03345	-0.12983	0.043535
Pedosphaera sp.	0.392142	0.064753	0.011451	-0.06564	-0.18035	0.036975

Table S5: Canonical correspondence analysis (CCA) for fungal communities. ANOVA table of the CCA model (A), Eigenvalues and their contribution to the scaled Chi-square (B). Scores for species in the first two components (highest scores are shown), species scaled proportioned to eigenvalues (C).

Table S5A:

ANOVA

	Df	ChiSquare	F	Pr(>F)
Model	6	0.63028	0.793	0.864

Table S5B:

Importance of components

	CCA1	CCA2	CCA3	CCA4	CCA5	CCA6
Eigenvalue	0.2657	0.11584	0.10115	0.06398	0.05351	0.0301
Proportion explained	0.1458	0.06358	0.05552	0.03512	0.02937	0.01652
Cumulative proportion	0.1458	0.20941	0.26492	0.30004	0.32941	0.34593

Table S5C:

Species scores

	CCA1	CCA2	CCA3	CCA4	CCA5	CCA6
Cryptococcus sp.	1.11445	0.369036	0.85219	0.206592	-0.3235	0.117307
Cladorrhinum sp.	0.72235	0.317987	-0.12432	-0.52805	0.7938	-0.4051
Penicillium citrinum	0.6278	0.204333	-0.07052	0.688437	-0.5441	-1.4151
Acrostalagmus luteoalbus	0.60276	-0.2674	0.760029	0.692359	0.3234	-0.25102
Cryptococcus fuscescens	0.56284	0.008541	0.26487	0.168223	0.1596	-0.59571
Neonectria sp.	0.5587	-0.25211	0.704434	0.653559	0.333	-0.19386
Tetracladium sp.	0.52137	-0.29803	0.507676	0.409709	0.1025	-0.23154
Coniolariella ershadii	0.5162	-0.01997	0.527921	0.385819	-0.0925	-0.09418
Chaetomium sp.	0.46664	-0.13988	0.66335	0.546768	0.2206	-0.28436
Metarhizium anisopliae	0.46504	0.345326	-0.52514	-0.5746	1.494	-0.48263
Fusarium oxysporum	0.40833	0.073049	0.058425	-0.32262	-0.116	0.131518
Humicola grisea	0.3833	0.378417	-0.02284	-0.24794	0.5765	-0.34542
Hansfordia sp.	0.37479	-0.05013	-0.41502	0.778428	0.8976	0.553268
Peniophora sp.	0.37108	-0.56695	0.50854	0.241481	0.1225	-0.06754
Acremonium blochii	0.34809	0.189951	0.25942	0.088453	0.2766	-0.00467
Calycina claroflava	0.3388	0.487644	0.48107	-0.13743	-0.474	0.838292
Penicillium nodositatum	0.3348	-0.1124	0.633723	-0.25831	-0.5275	-0.68092
Dactylonectria estremocensis	0.3228	-0.24038	0.065716	-0.67842	-0.4284	0.606488
Penicillium ochrochloron	0.27953	0.215827	-0.17022	-0.56202	0.3418	-1.05939
Holtermannia corniformis	0.27285	0.256185	0.420311	0.148273	-0.0208	-0.25956
Cryptococcus podzolicus	0.27124	0.162272	-0.15969	-0.12922	-0.0927	0.105592
Cephalotheca sulfurea	0.25635	-0.03941	-0.42956	0.111054	0.1225	-0.14599
Scytalidium cuboideum	0.25032	-0.17853	-0.33245	0.216152	0.5841	0.000858
Scytalidium sp.	0.20882	0.075278	-0.01714	0.124967	0.07061	0.192727

Cryptococcus aerius	0.19134	0.071912	-0.08592	0.260048	0.1436	-0.12786
Venturia hystrioides	0.1847	-0.02675	-0.06878	-0.40722	0.7753	-0.11931
Mortierella rishikesha	0.16504	-0.36623	-0.95523	-0.05554	-0.6483	-0.066
Arachnomyces gracilis	0.16501	-0.32828	-0.2074	0.836369	0.4482	0.170912
Aspergillus amstelodami	0.15459	-0.13999	-0.29212	0.590017	0.5816	0.54309
Lecanicillium sp.	0.1436	-0.88611	0.106287	-0.14634	-0.2268	0.210686
Acremonium persicinum	0.13505	0.303458	0.077052	-0.01712	-0.0489	-0.39946
Lophiostoma sp.	0.13158	0.000766	0.06781	-0.10491	-0.0726	0.030338
Clonostachys rosea	0.1299	0.271409	0.358792	-0.1997	0.05228	-0.22732
Truncatella laurocerasi	0.12811	0.17719	0.006564	-0.30468	0.4193	-0.14976
Ganoderma brownii	0.1161	-0.39575	0.096624	0.030226	0.5736	-0.04758
Mortierella amoeboidea	0.08273	-0.27787	-0.3866	-0.3853	-0.0929	0.279037
Cryptococcus phenolicus	0.06974	0.034382	0.076743	0.026955	9.59E-05	-0.07322
Aspergillus austroafricanus	0.06599	0.182446	-0.72365	0.771937	-0.3383	-0.52212
Veronaeopsis simplex	0.05568	-0.01419	-0.43392	-0.17827	0.191	-0.25153
Alternaria atra	0.04772	0.15823	0.08665	-0.1211	-0.1955	0.109611
Podospora sp.	0.04349	0.402698	0.047896	-0.04429	0.0452	-0.11608
Metacordyceps chlamydosporia	0.04242	0.191909	-0.29859	-0.37822	0.1638	-0.23786
Spiromastix warcupii	0.03242	-0.53109	-0.05011	-0.69589	-0.4098	0.103761
Penicillium menonorum	0.026	0.008825	0.063361	-0.08254	0.02202	-0.14104
Cladophialophora sp.	0.01671	0.262961	0.289349	-0.20773	-0.2603	0.664512
Rhodotorula lamellibrachiae	0.0166	0.134016	0.215551	0.567111	0.1564	0.056186
Aspergillus wentii	-0.0193	-0.5697	0.483928	0.032808	0.1285	0.454078
Cryptococcus terreus	-0.0301	-0.02635	0.016622	-0.02606	0.02417	-0.09928
Hypocrea atroviridis	-0.0341	0.43336	0.34215	-0.47482	-0.3226	-0.01758
Aspergillus piperis	-0.0542	-0.22666	0.581666	0.080866	0.1759	0.101951
